# Dr. Will McCaw

**Published:** 1911-12

**Authors:** 


Dr. Will McCaw of Geneva, died at Albuquerque, N. M., November 16, 1911. He was 48 years old and a graduate of P. and S. 1888.

